# Open Anterolateral Cordotomy for Cancer Pain: Indication, Efficacy, and Safety: A Systematic Literature Review

**DOI:** 10.3390/jcm15062111

**Published:** 2026-03-10

**Authors:** Edoardo Mazzucchi, Gianluca Galieri, Giuseppe La Rocca, Stefano Telera, Ilaria Monteferrante, Claudia Claroni, Domenico Policicchio, Adelina Amalia Ardelean, Giovanni Sabatino, Andrei Brinzeu

**Affiliations:** 1 IRCCS Regina Elena National Cancer Institute, 00144 Rome, Italy; stefano.telera@ifo.it (S.T.); ilaria.monteferrante@ifo.it (I.M.); claudia.claroni@ifo.it (C.C.); 2 Department of Neurosurgery, San Camillo Forlanini Hospital, 00152 Rome, Italy; gianluca.galieri@gmail.com; 3 Department of Neurosurgery, Università Cattolica del Sacro Cuore, 00168 Rome, Italy; giuseppe.larocca@policlinicogemelli.it (G.L.R.); giovanni.sabatino@policlinicogemelli.it (G.S.); 4 Department of Neurosurgery, Azienda Ospedaliero Universitaria “Renato Dulbecco” di Catanzaro, 88100 Catanzaro, Italy; dpolicicchio@aocz.it; 5 Department of Neurosurgery, University of Medicine and Pharmacy Victor Babes, 300041 Timisoara, Romania

**Keywords:** cancer pain, spinal cord, spinothalamic tract, palliative care, quality of life

## Abstract

**Background/Objectives**: Open anterolateral cordotomy (OALC) is a surgical intervention that has been performed to treat patients with persistent pain for more than a century. In recent decades, its application has been reduced in favor of other less invasive treatments. The present article aims to define indications, safety, and the efficacy profile of this procedure for the contemporary neurosurgeon. **Methods**: A systematic review of articles published from 2010 to 2025 has been performed. Only patients who underwent OALC for cancer pain were included. **Results**: Eleven articles were included in the systematic review for a total of 33 patients. Adequate pain response was obtained in 87.9% of cases. In 21.2% of patients, some kind of complication was reported, but they persisted only in three patients (9%). A single case of mirror pain was described. **Conclusions**: OALC is a procedure still performed in selected cases of persistent cancer pain with a favorable safety and efficacy profile.

## 1. Introduction

**Figure 1:**
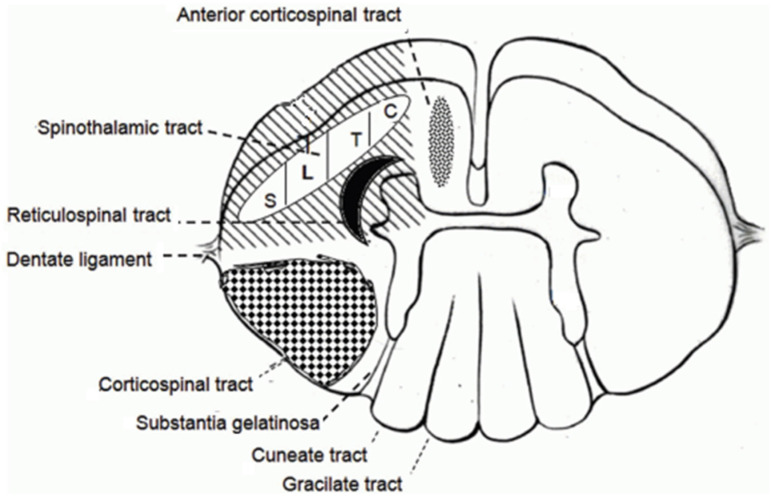
Schematic view of the thoracic cord. The diagonal-lined area depicts the lesion performed with an antero-lateral cordotomy. The somatotopic distribution of the spinothalamic tract is displayed. Abbreviations: C: cervical dermatomes; T: thoracic dermatomes; L: lumbar dermatomes; and S: sacral dermatomes.

Pain is a common symptom among patients suffering from oncologic disease; it is reported in about half of all the patients suffering from any type of cancer [1,2]. Moreover, it is frequently resistant to initial treatments, causing a marked worsening of quality of life. In the past, ablative procedures were largely performed for cancer pain due to the scarcity of alternatives. In recent decades, the use of more powerful and tolerated opioids and the growing diffusion of neuromodulation provided less invasive alternative treatments. Nevertheless, lesional treatment of pain is still performed in daily clinical practice when less invasive treatments fail or cause intolerable complications [3,4]. Main destructive procedures are cordotomy, trigeminal tractotomy, and the dorsal root entry zone lesion [3,4,5]. Cordotomy is one of the most studied among them, with a large number of articles and patients treated [3]. This term identifies the interruption of the spinothalamic tract in the anterolateral white matter of the spinal cord. The somatic nociceptive stimulus is transmitted by small myelinated delta A and unmyelinated C nerve fibers to the dorsal horn where a second-order neuron gives rise to the axons of the spinothalamic tract (Figure 1). The majority of these fibers decussates between 2 and 5 metamers above the level of the root where the nociceptive primary fiber entered the spinal cord to become part of the lateral spinothalamic tract with a somatotopic distribution [6,7,8]. This implies that anterolateral cordotomy must be performed on the opposite side and several metamers cranially than the upper limit of pain. The intervention was described for the first time in 1912 by Spiller and Martin as an open procedure [9], but the lesion may also be obtained with a percutaneous technique, as reported in 1965 by Mullan and colleagues [10]. In particular, in recent decades, the percutaneous fluoroscopy- or CT-guided [11] technique is the most performed one: it is, indeed, a less invasive approach that can be performed under local anesthesia with the patient awake, with immediate monitoring of the position of the electrode to rule out malposition and reduce the risk of post-operative motor impairment.

Nevertheless, open anterolateral cordotomy (OALC) is still practiced in selected cases [12]. Percutaneous cordotomy, as a matter of fact, is performed at a high-cervical level, where the risk of respiratory impairment is high in the case of bilateral pain, while a bilateral OALC is possible because the level of the lesion can be in the thoracic cord. Moreover, the awake procedure needs a collaborative patient who is able to lie still for a sufficient span of time to safely perform the procedure, thus excluding subjects affected by dementia, confusion, or anxiety.

A literature review by Raslan et al. [3] delineated the use of ablative procedures for cancer pain in 2011, with particular emphasis on the use of cordotomy. We performed a systematic review of articles published in the last 15 years, after the period analyzed by Raslan and colleagues. The purpose of this literature review was to define the current application of OALC and its indication, efficacy, and safety in the contemporary neurosurgical community.

## 2. Materials and Methods

A systematic literature review was performed according to the Preferred Reporting Items for Systematic Reviews and Meta-Analyses (PRISMA) 2020 guidelines [13,14] (see Supplementary Materials for the PRISMA checklists). A review protocol was registered in the International Prospective Register of Systematic Reviews (PROSPERO) database (ID 1114452, 28 July 2025).

The review questions were formulated according to the so-called PICO scheme, where P: patients; I: intervention; C: comparison; and O: outcomes. In particular, in patients with cancer pain (P), was OALC (I) efficient compared with other previous treatments (C) in obtaining satisfactory pain relief (O)? Pain relief is then defined as the primary outcome, while secondary outcomes are complications related to surgery and survival after OALC.

A search of articles regarding OALC in patients with cancer pain was conducted on Pubmed, Embase, Web of Science, and Scopus Databases with the following search terms ((cordotomy) OR (cordotomies) OR (“anterolateral cordotomy”) OR (ALC)) AND (cancer pain). The search was performed on 9 June 2025, filtering articles in English language published between 1 January 2010 and 1 June 2025. Congress abstracts, guidelines, reviews and articles regarding patients younger than 18 years old were excluded. Duplicate rejection was performed with the help of Zotero, Rayyan [15] and, subsequently, manually. The literature review was independently performed by two investigators (EM, GG). In cases of conflict, a third investigator resolved disputes (GS). The quality of evidence was investigated according to Joanna Briggs Institute Critical Appraisal Checklists for case series and for case reports [16,17].

## 3. Results

### 3.1. Selection of Articles

**Figure 2:**
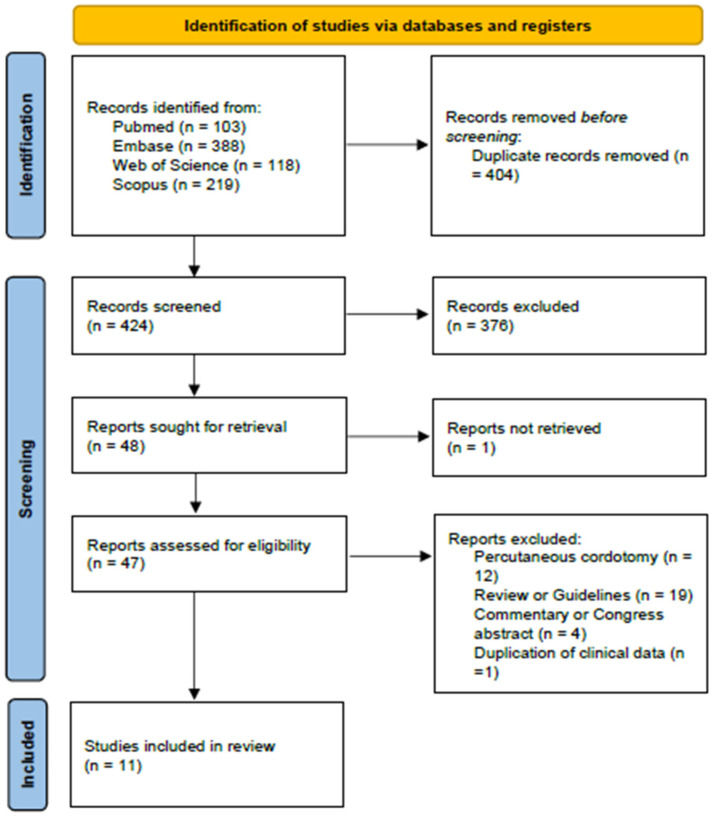
PRISMA flow chart for the identification of articles included in the systematic review.

A total of 828 papers were found in four different databases (Pubmed, Embase, Web of Science, and Scopus). Title and abstract screening was performed for the 424 articles resultant after the rejection of duplicates. Forty-eight full texts were subsequently carefully read [3,18,19,20,21,22,23,24,25,26,27,28,29,30,31,32,33,34,35,36,37,38,39,40,41,42,43,44,45,46,47,48,49,50,51,52,53,54,55,56,57,58,59,60,61,62,63,64] (see Figure 2 [13] for the criteria for exclusion), and 11 articles were included in the present review [18,21,22,33,34,35,38,40,50,54,58,60] (Table 1). All 11 articles were included after evaluation according to Joanna Briggs Institute Critical Appraisal Checklists for case series and for case reports [16,17]. Seven articles are case reports of one or two patients, while the others are case series with a cohort of 4 to 11 patients with cancer pain. The four case series [33,38,54,58] have been collected in a single center in Israel (two articles), the United Kingdom (one article), and United States of America (one article); the other articles, which are case reports, come from Australia (two), France (two), the USA, Canada, and Saudi Arabia.

### 3.2. Patient Population

Thirty-three patients underwent OALC for cancer pain in the selected articles. Three patients underwent bilateral thoracic OALC (9.1%) and 30 (90.9%) unilateral intervention. In 12 patients (36.4%) the operation was at the cervical cord, while thoracic cordotomy was performed in the other 21 (63.6%).

### 3.3. Indication for Surgery

**Figure 3:**
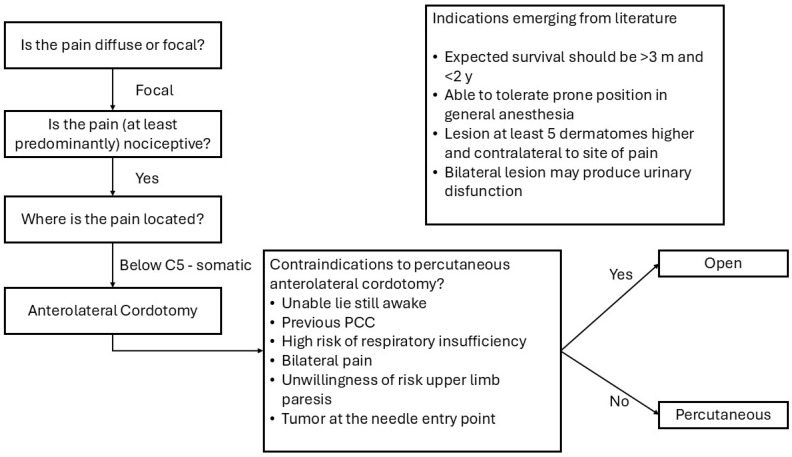
Flow chart of the indication for open anterolateral cordotomy. Abbreviations. PCC: Percutaneous Cervical Cordotomy; m: months; and y: years.

The indication for cordotomy was not clearly defined in most of the papers. According to three of the papers included in the review [8,33,54], the indication for anterolateral cordotomy was proposed for patients with focal somatic pain, at least predominantly nociceptive, because visceral neuropathic pain is thought to be resistant to anterolateral cordotomy [54]. Percutaneous procedure is preferable unless contraindicated. Contraindication to the less invasive approach may be the impossibility to lie still while awake for the procedure, high risk of respiratory insufficiency, necessity of a bilateral lesion, unwillingness to risk upper limb paresis, or having a tumor at the needle entry site (Figure 3). In the included patients, indication for OALC was mainly for pain involving the lower limb (16 patients, 48.5%) or hip (9 patients, 27.3%), while only six patients (18.2%) complained of pain in the arm or shoulder. Pain distribution including sacrum was reported in a patient who underwent bilateral thoracic OALC; the rib cage was involved in two patients who benefited from cervical OALC.

In five articles, the authors provided a justification for the choice of an open cordotomy instead of a percutaneous procedure (Table 1). The necessity of a bilateral cordotomy to treat pain was reported in five patients. In one case, the pre-existing cauda equina syndrome in a patient with pain involving the sacrum could be an adjunctive factor in favor of a bilateral procedure, as urinary retention (a possible complication of bilateral cordotomy) was already present. Compromised lung function was cited in one case. Moreover, reluctance to risk upper limb paresis (three patients), recurrence or failure after percutaneous cordotomy (two patients), anxiety for a percutaneous procedure, uncontrollable fear of needles, and impossibility of staying still while awake for the procedure (one patient for each indication) were considered significant warnings against the more diffuse and less invasive percutaneous intervention. In a single case, the presence of tumor at the site of entrance of the needle was an absolute contraindication for the procedure. Finally, one author raised a significant issue in palliative care medicine, declaring that one of the reasons for an OALC was the expertise for open cordotomy (and presumably the lack of expertise for percutaneous cordotomy).

### 3.4. Outcomes

**Figure 4:**
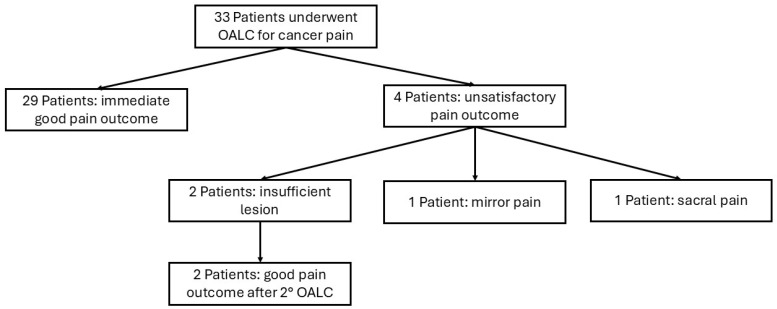
Flow chart of the pain outcome for patients included in the review. Abbreviations: OALC: open anterolateral cordotomy.

The definition of the pain outcome is not reported homogeneously in all papers. A Visual Analogic Scale or Numeric Rating Scale is applied in six papers [18,21,33,38,50,54]; Tomycz and colleagues used a subjective rating scale with five degrees [58], while the other four papers do not quantify the severity of pain before or after surgery. Moreover, what is considered a good pain outcome is clearly declared in only one paper [6]. Nevertheless, according to what is stated in the papers, an adequate pain outcome was reported in 29 patients (87.9%). In the other four patients, the failure was due to the occurrence of mirror pain (one patient), sacral pain (one patient), and insufficient lesion in two patients, who later obtained good pain control after re-operation (Figure 4). One patient experienced pain over the L5 dermatome distribution after bilateral OALC, but this was not considered sufficient to classify the outcome as a failure because of the relevant improvement of the other symptoms of the patient. In five of the articles included in this review, a reduction in opioid drugs is reported as an outcome measure [18,21,34,50,54]. Szylak and colleagues provided this information for only three of the five patients due to missing data. In all of these articles, the efficacy of the procedure led to a significant reduction or even withdrawal of these drugs, with a relevant reduction in the associated side effects.

Complications related to OALC were reported in seven patients (21.2%), but they were persistent in only three of them (9.1%).

The following complications were described: worsening of urinary function (three patients); persistent foot drop; worsening of pre-existent leg weakness; transient hemiparesis (resolved one month after surgery); and hypercalcemia with delirium. No case of post-surgical deafferentation pain was described among the patients included in the review. Survival of the patients after surgical intervention was highly variable and not always reported, so a statistical analysis cannot be performed. In the articles in which survival was reported, the duration of survival ranged from 3 weeks to 13 years.

## 4. Discussion

OALC has been reported in 33 patients between 2010 and 2025, with adequate pain control in 87.9% of patients and persistent complications in 9.1% of patients. The overall outcome of the procedure appears to be favorable considering the population of patients that are normally considered as candidates for this intervention. They are usually fragile patients with a relatively short life expectancy and failure of multiple (often invasive) treatments for a pain that severely deteriorated their quality of life.

### 4.1. Indication to Surgery

Classic indication to anterolateral cordotomy is the presence of unilateral nociceptive pain not responsive to less invasive treatment [3,22]. Nevertheless, as also demonstrated by this review, in selected cases, even bilateral pain may be addressed [21,34,58]. A bilateral lesion of the anterolateral cord at the cervical level is associated with an intolerable risk of respiratory insufficiency, while lesioning of the thoracic cord does not give rise to such complications [40]. This is a consequence of the anatomy of the spinal cord, because autonomic fibers that contribute to the respiratory drive lie in the anterolateral quadrant of the high cervical cord, while they are absent at the thoracic level [65,66,67]. Life expectancy is also considered a relevant factor for the choice of treatment in metastatic cancer patients [18]. On one hand, terminally ill patients are excluded due to the invasiveness of the procedure. On the other hand, when life expectancy exceeds 1–2 years, cordotomy is excluded, because it carries a risk or unsatisfactory pain relief that ranges from 13% in the early post-operative period (1–3 months) to 53% after 6 to 18 months [68]. As a consequence, the indication for OALC cannot be given by the neurosurgeon alone. In order to correctly identify this small subset of cancer pain patients, a multidisciplinary team that encompasses various treatment modalities [69] and is capable of carefully balancing the risks and benefits of the surgical intervention should collaborate to propose the most adequate treatment to these patients by carefully balancing risks and benefits. The OALC is indeed performed under general anesthesia with the patient in the prone position; this treatment option is therefore often discouraged by the labile general conditions of multi-metastatic patients in favor of procedures that are less demanding from an anesthetic point of view such as percutaneous procedures. This is the main reason why percutaneous cordotomy, which may be performed under local anesthesia, has replaced the “classic” open procedure in the majority of palliative care centers. This led OALC to be considered an abandoned procedure, rarely performed and even more rarely taught during the education of contemporary neurosurgeons. In 2003, Jones et al. [12] asked whether open cordotomy still had a role in cancer pain, demonstrating its utility in a series of selected cases. The indication for the intervention is of course reserved for a selected population of patients; this partly justifies the rarity of the intervention in the recent literature. But, in our opinion, we should not abandon this option. As a matter of fact, more than 20 years later, the present review shed light on the necessity of performing the open procedure in a specific subgroup of patients in which percutaneous cordotomy would not be possible.

Invasive treatments for cancer pain are applied in a small percentage of patients, even if 70–90% of cancer patients experience chronic pain [70] and if, in 30% of people with cancer pain, it is not controllable despite the best medical treatment [71]. It is probable that a significant part of this population does not benefit from the most indicated interventional cancer pain treatment due to inadequate access to healthcare. In particular, cancer pain patients are often unable to travel to distant, large volume centers, which can theoretically offer a wider range of treatment options. This literature review could be of help in bringing this procedure, which can also be replicated in small neurosurgical centers, to the attention of the medical and neurosurgical audience.

### 4.2. Surgical Technique

OALC is a relatively simple procedure that aims to interrupt the spinothalamic tract in the anterolateral white matter of the spinal cord. It was first described in 1912 by Spiller and Martin [9]; the procedure then evolved following the advancement of neurosurgery in general toward less invasive and safer treatments. A hemilaminectomy is described in three of the articles included in our review [38,54,58], and in one of these, a minimally invasive tubular-assisted approach is proposed [38]. The attempt to reduce the invasiveness of surgery appears particularly appropriate in these patients. As a matter of fact, multi-metastatic patients like the ones included in this review may have an increased risk of complications related to the less efficient healing of tissues after surgery as a consequence of previous radiotherapy or chemotherapy, comorbidities, and often prolonged treatments with corticosteroid drugs. Less invasive approaches are expected to reduce the length of incision and improve pain related to surgical treatment. However it should be noted that, in the article by Kimchi and colleagues, a second procedure was necessary due to inadequate pain control in two patients; since this second procedure obtained good pain outcome in both cases, it could be hypothesized that the unsatisfactory results of the first procedure were a consequence of the reduced exposure of the area to be lesioned, because no other retreatment has been reported with the standard open technique in the included articles.

### 4.3. Intraoperative Neurophysiological Monitoring

The use of Intraoperative Neurophysiological Monitoring (IONM) has become a standard of care during surgery for intramedullary tumors [72]. The main objective of IONM in OALC is the prevention of post-operative ipsilateral motor impairment, which has been reported to be as high as 22% [54,58]. In this review of the recent literature, four articles described the use of IONM [35,38,54,60]. More specifically, in addition to somatosensorial evoked potentials (SSEPs) and transcranial Motor Evoked Potentials (MEPs), direct monopolar stimulation of the spinal cord MEP is described in two papers. These initial reports suggest the potentiality of this technique in guiding the surgeon to a controlled lesion, thus avoiding the inadvertent section of the corticospinal tract. The impact of a post-operative motor impairment may be particularly relevant in this subset of patients with limited life expectancy. As a matter of fact, the patient may not have sufficient time to obtain significant improvement of motor functions with neurorehabilitation. Moreover, any effort to reduce the duration of hospitalization should be appreciated. Nevertheless, the absence of the possibility of performing any kind of IONM for OALC should not be considered, in our opinion, a limiting factor for surgeons who are planning to perform this procedure.

### 4.4. Mirror Pain

The appearance of mirror pain after cordotomy is a peculiar complication that gave the researchers the possibility of studying the complexity of the pain system [73], which still has to be clarified [74]. In this literature review, only one case (3%) of mirror pain has been reported, while an incidence between 33% and 73% can be found in the previous literature. This can be a consequence of the small patient sample included in the review. The largest case series and highest rates of new pain after cordotomy have been reported for the percutaneous procedure [75]. A direct comparison between percutaneous and OALC regarding this specific complication has not been made, to our knowledge, but it would be interesting to define if the different modalities in which the lesion is produced may influence the risk of mirror pain after surgery. In our opinion, future research could be able to improve our understanding of the pain system by studying this procedure in depth. For example, it could be possible to evaluate neurophysiology (e.g., laser evoked potential and somatosensorial evoked potentials) and functional neuroimaging before and after the lesion with the two techniques. Moreover, a clinical trial capable of making a direct comparison of clinical outcomes between the open and percutaneous techniques, with particular interest in mirror pain, would be desirable.

### 4.5. Reduction in Opioids Use

Opioids are largely used to treat pain in patients with cancer. Substantial evidence has associated the use and dosage of opioids administered with lower survival in cancer patients [76]. This might result from a bias of selection: the more serious the cancer diffusion, the higher the dose prescribed [77]. On the other hand, a growing body of the literature demonstrates a direct effect of morphine on cancer progression [78,79], immunosuppression [80], and response to treatment [81], which can explain a lower survival rate.

Unfortunately, in only five of the articles included in this review, a reduction in opioid drugs is reported as an outcome measure [18,21,34,50,54]. The efficacy of the procedure leads to a significant reduction or even withdrawal of these drugs, with relevant reductions in side effects that contribute to the improvement of the quality of life of this population of patients, apart from the possible effects on survival, which have been described.

### 4.6. Global Neurosurgery Perspectives

The present review does not aim to define the number of procedures for OALC performed in the world in the last 15 years. We wanted to understand the evolution of this historical intervention in the context of contemporary neurosurgery. If we consider the papers included, we find that the four case series [33,38,54,58] have been collected at a single center in Israel (two articles), the United Kingdom (one article), and the United States of America (one article) and the other articles, which are case reports, come from Australia (two), France (two), the USA, Canada, and Saudi Arabia. The distribution of the publications may reflect socio-economic conditions with different access to palliative medicine for cancer but also variable attitudes towards open surgery for cancer palliation according to social contexts and philosophic or religious beliefs. Aljuboori et al. [19] performed a comparative cost analysis in a specific context (USA) between percutaneous cordotomy and implantation of an intrathecal pump for the administration of analgesic drugs, stating that cordotomy is a cheaper yet equally effective procedure. In our opinion, a direct comparison is very difficult, since it should also take into account the effort of the patient who has to repeatedly reach the hospital where the pump must be recharged, with all the implications for a patient who is frequently terminally ill, while cordotomy is a single procedure, with a limited necessity for direct access to the hospital after the intervention. This should be even more carefully weighed in socio-economic and geographic contexts in which access to care may be very difficult from a logistic point of view. As a consequence, we wanted to highlight the importance of the diffusion of this procedure, especially in low–middle-income countries, where multiple trips to a palliative care center may be very difficult.

### 4.7. Limitations

This literature review has several limitations. First, the low level of evidence of the articles included, which are case reports or small case series, greatly limits the generalizability of the results. Nevertheless, we chose to include case reports in the literature review because of the rarity of the procedure in clinical practice, as reflected in the literature. Moreover, as previously pointed out, information regarding outcomes is reported partially and in a variety of modalities, thus further reducing the amount of data that can be evaluated. As a matter of fact, what is considered a good outcome is clearly declared in just one paper, pain is quantified in only seven of the articles, survival data are lacking in three, and a description of the evolution of complications over time is available in one.

## 5. Conclusions

Open anterolateral cordotomy is a surgical intervention still performed in selected cases of patients suffering from cancer-related pain. It showed a favorable risk–benefit ratio in a review of the literature of the last 15 years, but only articles with low levels of evidence are available. Future prospective studies with higher levels of evidence would be desirable to better delineate the efficacy and safety profile of this procedure.

## Figures and Tables

**Table 1:** Articles included in the review and clinical data collected from each article.

Author	Year	Study Type	Cohort Size	Technique	Pain Distribution	Indication to Open Procedure	Good Pain Outcome	Opioids	Failure	Complications	IONM	Survival
Atkin [21]	2010	CR	1	Bil OTC	Hip, sacrum, both legs	Complete cauda equina syndrome	100%	Reduced in 3 w	No	No	No	5 w
Tomycz [58]	2014	CS	4	Uni (1) or Bil (3) OTC	Hip (1), unilateral (2), or bilateral (1) legs	ND	75% (3/4)	ND	Mirror pain (1)	Foot drop (1), worsened leg weakness (1), transient leg weakness (1), worsened urinary function (2), mirror pain (1), hypotensive episode (1)	No	4 w–13 y
Jahangiri [35]	2015	CR	1	OCC	Left arm	Compromised lung function	100%	ND	No	No	Yes (SSEP + MEP)	>6 m
Hosking [34]	2015	CR	1	Bil OTC	Right hip	Expertise for open cordotomy; indication for bilateral cordotomy	100%	Reduced in 2 w	No	Urinary incontinence	No	3 m
Tsetsou [60]	2020	CR	2	OTC	Right leg	ND	100%	ND	No	Temporary hip weakness (1)	Yes (MEP)	ND
Hochberg [33]	2020	CS	5	OTC	Lower limb (unilateral)	Reluctance to risk upper limb paresis with PCC (3), fear of needles precluding awake PCC (1), tumor at the needle entry point (1)	80% (4/5)	ND	Sacral pain (1)	Hypercalcemia and delirium (1)	Yes	ND
Abdul-Razzak [18]	2022	CR	1	OTC	Right hip and leg	ND	100%	Reduced in 2 m	Contralateral pain (tumor progression	No	Yes	10 w
Leclerc [40]	2023	CR	1	OTC	Right hip	ND	100%	ND	No	No	ND	ND
Szylak [54]	2023	CS	5	Mon (4) or Bil (1) OTC	Unilateral hip (3), unilateral leg (1), bilateral legs (1)	Recurrence/failure after PCC (2), anxiety for PCC (1), unable to lie still awake (1), required bilateral cordotomy (1)	100%	Decreased (50%) at discharge (3 patients)	Unilateral L5 pain after bilateral cordotomy (1)	No	Yes (SSEP + MEP + SpMEP)	2–10 m
Seznec [50]	2024	CR	1	OTC	Left hip	ND	100%	Withdrawal at 6 m	No	No	No	>6 m
Kimchi [38]	2025	CS	11	CTAC	Unilateral arm (3), unilateral shoulder (2), rib cage (2), unilateral leg (2)	ND	82% (9/11)	ND	Reoperation for inadequate pain control (2)	Transient hemiparesis (1)	Yes (SSEP + MEP)	3 w (1); 9 m (1); >1 y (7)

Berger (2020) [22] seems to report the same case series of Hochberg (2020) [33]. Abbreviations: IONM = Intraoperative Neurophysiological Monitoring; CR = case report; Bil = bilateral; OTC = Open Thoracic Cordotomy; w = week; CS = case series; Uni = unilateral; ND = Not Declared; y = year; OCC = Open Cervical Cordotomy; SSEP = somatosensorial evoked potentials; MEP = Motor Evoked Potentials; m = months; PCC = Percutaneous Cervical Cordotomy; SpMEP = Spinal Cord Motor Evoked Potentials; and CTAC = Cervical Tubular Assisted Cordotomy.

## Data Availability

No new data were created or analyzed in this study. Data sharing is not applicable to this article.

## Supplementary Materials

The following supporting information can be downloaded at: https://www.mdpi.com/article/10.3390/jcm15062111/s1. Ref. [13].

## Abbreviations

The following abbreviations are used in this manuscript:

OALC	Open Anterolateral Cordotomy
CT	Computed Tomography
IONM	Intra-Operative Neurophysiological Monitoring
OTC	Open Thoracic Cordotomy
OCC	Open Cervical Cordotomy
CR	Case Report
CS	Case Series
ND	Not Declared
CTAC	Cervical Tubular Assisted Cordotomy
SSEP	Somato-Sensorial Evoked Potentials
MEP	Motor Evoked Potentials
SpMEP	Spinal Cord Motor Evoked Potentials
PCC	Percutaneous Cervical Cordotomy
